# The structural characteristics of the lifestyle among older adults and its impact on the health in China

**DOI:** 10.3389/fpubh.2023.1286530

**Published:** 2023-12-13

**Authors:** Xiaohui Wang, Haimin Miao, Qiaosen Jin

**Affiliations:** ^1^School of Public Administration, Yanshan University, Qinhuangdao, China; ^2^School of Economics and Management, Yanshan University, Qinhuangdao, China; ^3^Center for Social Security Studies of Wuhan University, Wuhan, China

**Keywords:** lifestyle, older adults, health, healthy aging, China

## Abstract

**Objective:**

Enhancing overall health remains a primary global objective, with lifestyle being a crucial factor impacting the health status among older adults. This study focuses on the lifestyle of Chinese older adults, systematically exploring the evolution and characteristics of their lifestyle and investigating its impact on their health.

**Methods:**

Utilizing panel data from the Chinese Longitudinal Health Longevity Survey (CLHLS) from 2008, 2011, 2014, and 2018, we adopted 15 indicators reflecting older adults’ lifestyle. Latent class analysis and ordinary least square regression were used to uncover the structural nuances of the Chinese older adults’ lifestyle and its effects on health.

**Results:**

The study identifies three distinct lifestyle categories for the older adults: relatively positive, mixed, and relatively negative. It reveals that the Chinese older adults do not predominantly adopt a positive lifestyle, and this trend persisted from 2008 to 2018. Each improvement in lifestyle category significantly boosts the older adults’ physical health, mental health, and self-assessed health by 11.2%, 3.6%, and 17.1%, respectively.

**Conclusion:**

To attain the strategic aim of healthy aging, efforts should prioritize encouraging the older adults to adopt a positive lifestyle, enhance the geriatric health service system, and promote an intelligent lifestyle management model.

## 1 Introduction

Health is the most fundamental human right, and the achievement of a high health standard stands as one of the most vital global social goals. The World Health Organization pointed out that health is a state of complete physical, mental and social well-being and not merely the presence of disease or uncertainty, emphasizing the multidimensional nature of health and the need to assess the health status of individuals or groups from multiple aspects. However, older adults generally have varying degrees of health problems. In China, nearly 70% of older adults in China suffer from chronic diseases, the proportion of disabled and semi disabled older adults in the population of older adults has reached 18.3, and 80–90% of older adults have varying degrees of psychological problems, personality disorders, or bad behavior habits. Among them, about 27% of older adults have psychological problems such as anxiety and depression that affect their normal physical and mental health (1).

Research on influencing factors of health can be traced back to the to the last century, Grossman (2) pointed out that healthcare, income level, lifestyle, education level, and living environment are all related to health levels. As a response to the global aging phenomenon, scholars have analyzed the impact of various factors the health status of the older adults from multiple perspectives such as medicine, sociology, economics, and management. Studies reveal that individual demographic attributes such as marital status, income, educational attainment, and socioeconomic status are crucial variables that significantly impact older adults’ health (3, 4). Family characteristics such as social support, residential arrangements, or older adults’ care patterns have a positive impact on the physical and mental health of the older adults (2, 5, 6). Social characteristics such as social insurance and medical care services are also related to the health level of the older adults (7, 8).

In the past 20 years, more and more people have been prone to chronic diseases related to lifestyle. Lifestyle related to health have gradually become a research hotspot. According to data from the World Health Organization, health = 15% genetic factors + 10% social factors + 8% medical conditions + 7% climate conditions + 60% personal behavior and lifestyle factors (9). Lifestyle has become the primary factor restricting human health. Compared with other age groups, the physical fitness and activity abilities of the older adults are relatively weak, and they are deeply influenced by historical culture and traditional customs. The lifestyle of the older adults has its own uniqueness. In recent years, research on the lifestyle of the older adults has mainly focused on two aspects. On the one hand, the current situation and determining factors of lifestyle and health knowledge among the older adults. Yin et al. (10) described the lifestyle and health knowledge status of older adults through sampling methods, and pointed out that their health literacy and behavior are influenced by education level, race, previous occupation, family income, age, physical exercise, physical examination, smoking, and access to health information. Warr et al. (11) pointed out that socioeconomic status often affects individual lifestyle through the combined effect of material, neighborhood environment or social psychological factors. In recent years, the Internet has provided new tools for older adults to actively obtain health information and learn about disease prevention knowledge (12, 13). On the other hand, the impact of older adults’ lifestyle on their health status. Research has shown that a healthy lifestyle plays a significant role in effectively reducing the incidence of diseases such as coronary heart disease and obesity, delaying aging, and improving physical function. Specifically, Miranda et al. (14) found that physical exercise and reasonable dietary adjustments can alleviate symptoms in hypertensive patients. Ersserc et al. (15) pointed out that lower sleep quality not only affects human health but also hinders the recovery of diseases in the older adults. Karim et al. (16) found a significant positive correlation between physical activity and health self-assessment in 15 European Union countries. Room and Babor (17) pointed out that drinking alcohol can cause over 60 health threatening diseases and life threatening events, accounting for 4% of the global disease burden. Joana et al. (18) found that physical activity during leisure time significantly affects self-assessed health status, but alcohol consumption is not associated with poor self-assessed health and Zhu et al. (19) found physical activity has a negative predictive effect on geriatric depression. Helgeson (20) pointed out that communication and interaction between neighbors and friends can alleviate the loneliness and psychological pressure of the older adults, and have a positive impact on their health self-assessment. Gupta and Yang et al. pointed out that social activities play an important role in promoting the health of the older adults (21, 22). Cotte et al. and Chopik found that internet use significantly reduces loneliness and depression of older adults (23, 24) and Zhao et al. (25) found WeChat use is associated with a lower level of depression.

Existing literature reveals a substantial body of research investigating the health issues of the older adults, employing various indicators. Nonetheless, the overall body of work exploring the correlation between lifestyle and older adults’ health remains somewhat scant, manifesting several distinct shortcomings: Firstly, the preponderance of existing studies fixates on how risky lifestyle impacts the physical health of the older adults, as well as the how social participatory lifestyle impacts on their mental health. This focus overlooks the comprehensive value of lifestyle for health, as regardless of which lifestyle, it is closely related to physical health and mental states. Secondly, many investigations choose a single behavior as the principal dependent variable, often utilizing data from a specific region. Even in studies featuring multidimensional indicators, the treatment of lifestyle variables typically amounts to a simplified summary, which fails to fully capture the intricate effects of the lifestyle of Chinese older adults on their health. Thirdly, given the profound connection between health problems and medicine, most of the existing research adopts a medical paradigm (26), with fewer studies adopting the perspectives and methodologies of economics or demography. Consequently, there is a notable absence of discussions surrounding endogenous problems and intermediary influence mechanisms.

Building on these considerations, based on the four-phase data from the Chinese Longitudinal Health Longevity Survey, this study contributes to the existing body of knowledge in significant ways. Through latent class analysis, strive to systematically illustrate the broad panorama of the older adults’ lifestyle by incorporating 15 lifestyle indicators. Through ordinary least square, assess the impact of older adults lifestyle on their physical health, psychological health, and self-perceived health, and delve into potential endogenous issues associated with the lifestyle’s impact on older adults’ health, thereby enhancing and supplementing existing research. The study aims to reveal the overall picture of the lifestyle of the older adults, comprehensively examine the impact of their lifestyle on the health, thus promoting the cultivation of healthy lifestyle for the older adults, improving their health level, and facilitating the achievement of active aging.

## 2 Methods

### 2.1 Study design and sample

The data utilized in this study is derived from the Chinese Longitudinal Health Longevity Survey (CLHLS), a project collaboratively undertaken by Duke University and Peking University. The CLHLS is recognized as the largest global survey in the field of health and longevity, specifically focused on China. To date, CLHLS has conducted face-to-face interviews in 1998, 2000, 2002, 2005, 2008, 2011, 2014, and 2017–2018, with 8,959, 11,161, 20,428, 18,549, 20,366, 10,188, 7,192, and 15,874 individuals, respectively by using multi-stage hierarchical clustering sampling. The sample encompasses the eastern, central, western, and northeastern regions of mainland China, covering 23 provincial administrative units. The survey extensively investigates the health status and social, behavioral, biological, and environmental risk factors of the older adults. In addition to baseline surveys of those aged 80 years and above, earlier surveys have incorporated those aged 65 years and above, evaluating diverse facets such as their basic personal and familial statuses, socio-economic backgrounds, family structures, economic sources, health conditions, self-evaluations of life quality, cognitive functions, psychological characteristics, daily activity capabilities, lifestyle patterns, care provision, disease treatments, and medical expenditures.

Given variations in the age range and specific contents of prior respondents, this study excludes the initial three periods of data and employs CLHLS data from 2008, 2011, 2014, and 2018. We establish a balanced panel data using ‘ID’ as the sole matching criterion. Such panel data not only depicts the dynamic changes in lifestyle and health status among China’s older adults, but also addresses the interference of some time-invariant factors on dependent variables. It enables us to handle the missing variable problem more effectively and derive more robust causal inferences. After excluding missing values of required variables, our sample comprises a total of 2,454 older adults.

### 2.2 Variables

#### 2.2.1 Dependent variable

This study measures the health status of the older adults from three perspectives: physical health, psychological health, and self-assessed health.

##### 2.2.1.1 Physical health

Physical health is evaluated using the Activities of Daily Living (ADL) scale, a widely utilized tool that reflects the level of disability in older adults. The scale comprises six indicators: dressing, bathing, eating, getting out of bed, using the bathroom, and walking (27). If all six activities can be performed independently, as per the questionnaire response ‘no need for any help’, the ADL score is considered unimpaired, and is assigned a value of 4. Conversely, if assistance or complete dependence is required for at least one activity, as indicated by the responses “need help” or “cannot do it at all,” this denotes disability. Specifically, 1–2 impaired items represent mild disability, 3–4 moderate disability, and 5–6 severe disability, assigned respective values of 3, 2, and 1.

##### 2.2.1.2 Psychological health

The CLHLS questionnaire was only designed in 2018 based on Andresen’s Short Version of Center for Epidermal Studies Depression Scale (CES-D-10) and Generalized Anxiety Disorder (GAD-7) to reflect depressive and anxiety symptoms in the older adults. However, in previous surveys, only personality and emotional characteristics were inquired about. Analyzing existing research and commonly used depression screening scales, it can be found that anxiety and tension are one of the important indicators for measuring an individual’s mental health status. Due to questionnaire constraints, the study uses the question ‘Do you often feel fearful and anxious ‘to reflect the psychological health of the participants. The responses “always,” “often,” “sometimes,” “rarely,” and “never” are assigned values of 1, 2, 3, 4, and 5, respectively. These values correspond to varying states of psychological health, ranging from very poor to very good.

##### 2.2.1.3 Self-assessed health

Self-assessed health offers an individual’s evaluation of their own physical condition. It has been demonstrated in research that self-assessments can capture comprehensive health information from various aspects such as physical, psychological, and social well-being. It has good consistency with the subjective and objective health status of the body, and is more stable than doctors’ estimated health status (28, 29). This study selects the “How do you feel about your current health status” in the questionnaire, with a value of 1–5, representing very bad, not good, average, good, and very good, respectively.

#### 2.2.2 Independent variable

The lifestyle related to health that this study focuses on is a set of health-related behaviors that people choose based on life opportunities under existing conditions (30), including smoking, drinking, eating, rest, exercise, physical examination, social interaction, and other aspects of life. According to policies such as the Action Plan for a Healthy Lifestyle for All and existing research on health-related lifestyle (31, 32), this study employs 15 indicators to capture the lifestyle aspects of the older adults, encompassing four primary domains: daily diet, personal hygiene, physical activity, and leisure activities. The daily diet indicators consider the consumption frequency of fresh fruits, fresh vegetables, pickled vegetables, sugar or candy, along with tea-drinking habits, and the long-term preference for drinking water type (boiled or un-boiled). Personal hygiene indicators encompass smoking and alcohol consumption behaviors and the adequacy of sleep (specified as 7 to less than 9 h per day). Physical activity indicators include the engagement in fundamental daily exercises such as dancing, fitness, and qigong; the frequency of partaking in outdoor personal activities; and the frequency of gardening and pet care activities, such as raising cats and dogs, which are also classified under personal sports. For instance, walking dogs necessitates going outside for a walk. Leisure activities indicators capture the frequency of watching television or listening to the radio—significant means for the older adults to stay informed about current events, which forms the foundation for societal participation and personal opinion shaping. Other considerations include the frequency of card or mahjong games, prevalent leisure activities, and the frequency of participation in organized events managed by communities or other entities, such as choir competitions and sports events.

#### 2.2.3 Control variables

Gender, registered residence, age, educational level, social security participation, occupation before the age of 60, income level, living arrangements, and marital status are listed as the control variables in this study.

### 2.3 Measurement model

#### 2.3.1 Latent class analysis

The lifestyle of the older adults covers a wide range, with differences among different variables and different statistical methods for various types of data. Therefore, this study uses latent category analysis to identify potential categories of older adults’ lifestyle based on the data itself. Latent Class Analysis (LCA) is the process of interpreting the associations between explicit variables of individuals, determining their potential feature classification, determining the proportion of different types of population, and then adopting different intervention strategies for the segmented population. The basic assumption is that the probability distribution of various responses to explicit variables can be explained by a few mutually exclusive latent category variables, and each category has a specific tendency to choose responses to each explicit variable (33). This study uses Mplus7.4 software to conduct latent category analysis on 15 explicit variables of older adults’ lifestyle. Firstly, find the best model by comparing the fitting indicators of each model. In terms of setting the fitting evaluation indicators for the model, gradually increase the number of categories from the zero model with only one category, and calculate the *p*-values of AIC, BIC, aBIC, Entropy index, LMR, and BLRT. The smaller the values of AIC, BIC, and aBIC, the higher the fit of the model. The Entropy index ranges from 0 to 1, closer to 1, and the more accurate the model classification is. The *p* values of LMR and BLRT reach a significant level, indicating that the model in this category is significantly better than the model in the previous category. Secondly, according to conditional probability, we can judge the tendency of each category and name the potential types of health behaviors; Finally, the Bayesian posterior probability is used to infer the potential category of the older adults, and then the health behavior of each older adult is scientifically classified.

#### 2.3.2 Ordinary least square

Given that the three variables of physical health, mental health, and self-evaluation health are all ordered variables in the indicators for measuring the health status of older adults, some scholars in existing literature often use ordered probability models (Ordered Logit) or least squares (OLS) for estimation. However, studies have shown that when the model is set correctly, there is no difference between the advantages and disadvantages of OLS and Ordered Logit models (34). However, the estimated coefficient of Ordered Logit model has no clear meaning and does not have the meaning of marginal utility. Therefore, this study regards the health status of the older adults as a specific value in the process of empirical analysis and uses panel data linear regression to estimate. Using the software of Stata 16.0, this study adopts both fixed effects and random effects models, and selects them through the Hausman test. To further explore the impact of lifestyle categories on the health status of the older adults, the following model is constructed.

(1) $$\mathrm{HRQO}L_{\mathrm{it}}=\alpha +\beta_{1}\mathrm{Life}style_{\mathrm{it}}+\beta X_{\mathrm{it}}+\gamma Z_{i}+\eta_{i}+\varepsilon_{\mathrm{it}}$$

Among them, i represents the older adult individual, t represents the observation time point, $\mathrm{HRQO}L_{\mathrm{it}}$ represents the health status of older adult individual i at time point t , $\mathrm{Lifestyl}e_{\mathrm{it}}$ represents the lifestyle category of older adult individual i at time point t , $X_{\mathrm{it}}$ 、 $Z_{i}$ is the control variable that changes at each observation time point and the independent variable that does not change at different time points, including gender, registered residence, age, education level, social security participation status, occupation before the age of 60, income level, living arrangements, marital status, $\eta_{i}$ is a random individual effect, random term $\varepsilon_{\mathrm{it}}$ is the Error term of older adult individual i at different time points.

## 3 Results

### 3.1 Descriptive analysis of lifestyle among older adults

Whether it is dietary habits, exercise, or even leisure activities, older adults exhibit unique lifestyle characteristics, which reflect the general patterns of their later life and reveal the impact of various factors on their lifestyle. With the development of society, the lifestyle of the older adults has also been slowly changing over the past decade, and the distribution of various lifestyle indicators varies. The specific indicators are shown in Table 1.

**Table 1:** Statistical table of healthy lifestyle indicators for the older adults (*n* = 2,454).

Category	Life indicator	Measurement	2008 (%)	2011 (%)	2014 (%)	2018 (%)
Daily diet	Fresh fruit	Rarely or never	19.93	24.12	24.16	24.69
		Occasionally	37.65	36.15	34.60	34.11
		Quite often	30.68	25.06	26.32	23.47
		Everyday or almost everyday	11.74	14.67	14.91	17.73
	Fresh vegetables	Rarely or never	1.10	1.26	2.00	2.93
		Occasionally	7.09	5.09	6.28	8.03
		Quite often	25.51	28.73	28.97	26.16
		Everyday or almost everyday	66.30	64.91	62.75	62.88
	Salt-preserved vegetables	Rarely or never	31.66	35.62	37.25	45.64
		Not every month, but occasionally	15.77	16.18	15.81	16.26
		Not every week, but at least once per month	10.19	9.90	12.10	11.21
		Not every day, but at least once per week	18.58	20.86	19.40	16.87
		Almost everyday	23.80	17.44	15.44	10.02
	Sugar	Rarely or never	33.74	37.69	38.51	45.68
		Not every month, but occasionally	24.04	19.36	20.33	16.50
		Not every week, but at least once per month	11.29	11.90	12.06	11.04
		Not every day, but at least once per week	19.60	19.44	18.99	16.99
		Almost everyday	11.33	11.61	10.11	9.78
	Tea	Rarely or never	48.78	55.38	63.08	74.82
		Not every month, but occasionally	7.66	6.52	4.77	2.77
		Not every week, but at least once per month	2.61	2.93	2.77	1.43
		Not every day, but at least once per week	5.66	7.13	6.56	4.69
		Almost everyday	35.29	28.04	22.82	16.30
	Water	Un-boiled water	4.69	7.01	5.18	7.95
		Boiled water	95.31	92.99	94.82	92.05
Personal hygiene	Smoke	No	77.42	80.03	81.87	84.47
		Yes	22.58	19.97	18.13	15.53
	Drink	No	77.91	79.50	82.36	85.74
		Yes	22.09	20.50	17.64	14.26
	Sleep	Too much or too little	55.62	57.82	59.09	59.62
		Normal	44.38	42.18	40.91	40.38
Exercise	Do exercises	No	65.28	56.97	63.57	69.11
		Yes	34.72	43.03	36.43	30.89
	Outdoor activities	Rarely or never	25.06	24.98	27.38	31.54
		Not every month, but occasionally	5.26	5.30	5.46	7.42
		Not every week, but at least once per month	4.32	2.40	2.44	4.44
		Not every day, but at least once per week	10.55	11.33	11.57	15.65
		Almost everyday	54.81	55.99	53.14	40.95
	Garden work	Rarely or never	79.67	70.62	72.29	83.09
		Not every month, but occasionally	2.81	2.77	2.77	1.75
		Not every week, but at least once per month	1.47	1.51	1.43	0.98
		Not every day, but at least once per week	2.16	3.59	3.75	2.20
		Almost everyday	13.90	21.52	19.76	11.98
Leisure activities	Watch TV and/or listen to radio	Rarely or never	11.86	14.59	15.57	26.00
		Not every month, but occasionally	4.20	3.30	3.71	3.38
		Not every week, but at least once per month	3.87	2.81	2.53	2.97
		Not every day, but at least once per week	10.64	9.94	11.04	10.55
		Almost everyday	69.44	69.36	67.16	57.09
	Paly cards and/or mah-jong	Rarely or never	77.10	78.24	78.48	84.80
		Not every month, but occasionally	5.26	4.36	4.65	2.44
		Not every week, but at least once per month	3.34	2.08	2.93	1.55
		Not every day, but at least once per week	5.62	5.99	5.05	4.85
		Almost everyday	8.68	9.33	8.88	6.36
	Social activities (organized)	Rarely or never	81.21	79.54	80.28	88.30
		Not every month, but occasionally	7.29	10.02	9.86	5.42
		Not every week, but at least once per month	4.12	2.81	2.77	1.83
		Not every day, but at least once per week	2.61	2.81	2.89	1.71
		Almost everyday	4.77	4.81	4.20	2.73

From the various indicators of daily diet, older adults do not have the habit of consuming fresh fruits, but their consumption of fresh vegetables is better, and their preference for pickled foods, sweets, and tea is decreasing. The vast majority of older adults are accustomed to drinking boiled water. From various indicators of personal hygiene, the vast majority of older adults do not have the habit of smoking and drinking, but their sleep quality is poor. From the perspective of various indicators of exercise, the older adults’ form of exercise is mainly outdoor activities, supplemented by daily physical exercise, and having fun growing flowers, plants, and pets. About 70% of older adults do not have the habit of daily exercise. From the various indicators of leisure activities, the enthusiasm of older adults for leisure activities during the past decade was not high. The vast majority only learned about current events through television and radio, and did not actively participate in collective activities such as playing cards or mahjong, as well as organized social activities.

### 3.2 Results of LCA on lifestyle among the older adults

The LCA model suitability indicators for the four survey periods from 2008 to 2018 are shown in Table 2. The smaller the values of AIC, BIC, and aBIC, the higher the fit of the model; The Entropy index reflects the accuracy of classification, with a range of 0–1. The closer the value is to 1, the more accurate the model classification is. When Entropy is equal to 0.6, it indicates that about 20% of individuals have classification errors. When Entropy’s value is about 0.8, the accuracy of classification reaches over 90%; LMR and BLRT reflect the fitting differences of potential category models. If the *p*-values of the two indicators reach a significant level, it indicates that the model of this category is significantly superior to the model of the previous category. By observing the *p*-values of AIC, BIC, aBIC, LMR, and BLRT in each model, and comparing them comprehensively, the three categories are the most ideal models. Therefore, this study divides the lifestyle of the older adults into three categories.

**Table 2:** Summary of LCA model adaptability indicators.

Year	Model	AIC	BIC	aBIC	Entropy	LMR(p)	BLRT(p)
2008	1	66168.093	66417.729	66281.108	1.000	--	--
	2	64931.779	65436.855	65160.435	0.610	0.0000	0.0000
	3	64585.328	65345.845	64929.627	0.670	0.0004	0.0000
	4	64255.744	65271.702	64715.685	0.675	0.7747	0.0000
	5	64243.067	65514.466	64818.650	0.735	0.8382	0.0741
2011	1	66594.718	66844.354	66707.733	1.000	--	--
	2	65317.553	65822.629	65546.209	0.608	0.0000	0.0000
	3	64976.938	65737.456	65321.237	0.604	0.0008	0.0000
	4	64776.061	65792.019	65236.002	0.588	0.7742	0.0000
	5	64591.252	65862.651	65166.836	0.617	0.8349	0.0000
2014	1	65733.217	65982.853	65846.232	1.000	--	--
	2	64172.352	64677.429	64401.009	0.655	0.0000	0.0000
	3	63796.510	64557.027	64140.808	0.635	0.0390	0.0000
	4	63541.938	64557.896	64001.879	0.661	0.7647	0.0000
	5	--	--	--	0.643	--	--
2018	1	61946.611	62196.247	62059.626	1.000	--	--
	2	60327.489	60832.565	60556.145	0.663	0.0000	0.0000
	3	59796.900	60557.417	60141.199	0.793	0.5709	0.0000
	4	59528.608	60544.566	59988.549	0.703	0.7672	0.0000
	5	59345.141	60616.540	59920.725	0.697	0.7569	0.0000

By comprehensively observing the conditional probabilities of various lifestyle indicators under different options, the lifestyle of the older adults can be divided into three categories of relatively positive, mixed, and relatively negative. The potential category probabilities of older adults’ lifestyle in each year are shown in Table 3. In 2008, the lifestyle of the older adults was mainly relatively positive, but the probability of potential categories was only 53.87. However, as age increases, the lifestyle of the older adults has not shifted toward a healthier lifestyle. On the contrary, the potential category probability of a relatively positive lifestyle has decreased. In 2018, the potential category probabilities of a relatively positive, mixed, and relatively negative lifestyle were 45.40%, 2.69%, and 51.92%, respectively.

**Table 3:** Probability of potential categories of healthy lifestyle for the older adults.

Category	2008	2011	2014	2018
Relatively positive	53.87%	39.98%	40.59%	45.40%
Mixed	9.50%	20.21%	24.25%	2.69%
Relatively Negative lifestyle	36.63%	39.81%	35.17%	51.92%

### 3.3 Results of OLS

According to the potential category analysis above, the lifestyle of the older adults is divided into negative lifestyle, mixed lifestyle, and positive lifestyle, with values assigned as 1, 2, and 3, and included in the model (1). Table 4 reports the estimated results of the lifestyle categories of older adults on physical health, mental health, and self-evaluation health. Since the fixed effect can only estimate the coefficient of independent variable that changes with time, and the variable that does not change with time will be discarded in the model, the results of the four variables of gender, registered residence, education level and occupation are not output. From the Hausman test results, it can be seen that the estimation results of the three models are suitable for analysis using the FE model.

**Table 4:** Results of the impact of lifestyle categories on the health of the older adults.

Variable		Physical health		Mental health		Self-assessed health	
		FE	RE	FE	RE	FE	RE
Core variable	Lifestyle	0.112^***^ (0.013)	0.118^***^ (0.010)	0.036^*^ (0.019)	0.085^***^ (0.015)	0.171^***^ (0.015)	0.205^***^ (0.013)
Control variables	Age	−0.052 (0.039)	−0.014^***^ (0.001)	0.112^***^ (0.039)	−0.010^***^ (0.002)	−0.011 (0.033)	−0.004^**^ (0.002)
	Gender	-	0.038^*^ (0.019)	-	0.043 (0.029)	-	0.081^***^ (0.029)
	Residence	-	−0.045^**^ (0.020)	-	0.077^***^ (0.029)	-	−0.023 (0.029)
	Education	-	−0.001 (0.003)	-	0.004 (0.004)	-	0.000 (0.004)
	Occupation	-	0.003 (0.007)	-	−0.037^***^ (0.011)	-	0.019 (0.011)
	Social Security	0.004 (0.032)	0.028 (0.029)	0.082^*^ (0.049)	0.038 (0.041)	−0.039 (0.039)	0.009 (0.034)
	Living	0.212^***^ (0.044)	0.157^***^ (0.031)	0.224^***^ (0.048)	0.161^***^ (0.035)	0.356^***^ (0.046)	0.263^***^ (0.036)
	Marriage	0.182^***^ (0.046)	0.103^***^ (0.023)	0.301^***^ (0.060)	0.218^***^ (0.032)	0.336^***^ (0.051)	0.203^***^ (0.032)
	Income	0.018^**^ (0.009)	0.018^***^ (0.007)	−0.001 (0.011)	0.028^***^ (0.009)	0.049^***^ (0.01)	0.063^***^ (0.008)
Constants		7.193^**^ (2.975)	4.432^***^ (0.138)	−4.945^***^ (2.927)	4.094^***^ (0.199)	3.003 (2.506)	2.322^***^ (0.190)
Year dummy		Yes	Yes	Yes	Yes	Yes	Yes
Within R2		0.044	0.046	0.534	0.532	0.070	0.066
Sample size		9,816	9,816	9,816	9,816	9,816	9,816
Hausman test [*p*-value]		17.82 [0.0067]		58.17 [0.0000]		69.87 [0.0000]	

The data in parentheses represents robust standard error. ^*^p < 0.1, ^**^p < 0.05, ^***^p < 0.01. The same below.

From the estimation results, lifestyle categories have a significant positive impact on the physical health of the older adults (coefficient 0.112, *p* < 0.01). For each category of lifestyle improvement, the physical health of the older adults will increase by 11.2%. The impact of lifestyle categories on the mental health of the older adults is also positive (coefficient 0.036, *p* < 0.1), indicating that for each category of lifestyle improvement, the mental health of the older adults will increase by 3.6%. At the same time, the lifestyle category significantly improved the self-assessed health of the older adults (coefficient 0.171, *p* < 0.01), meaning that for each category of lifestyle improvement, the psychological health of the older adults will increase by 17.1%. Overall, a good lifestyle can improve the health status of the older adults. This indicates that the comprehensive effects of daily diet, personal hygiene, exercise, and leisure activities on the quality of life related health of the older adults are significant, which are closely related to family economic status and community services.

## 4 Discussion

This study enhances the current dimensions of research into older adults’ health status by using panel data drawn from individual microdata from the fourth phase of the survey on the influencing factors of health and longevity of the older adults in China. The study selected 15 items to represent aspects of daily diet, personal hygiene, exercise, and leisure activities, which together comprise a four-dimensional view of an older adult’ lifestyle. The fundamental and structural characteristics of the lifestyle of Chinese seniors were revealed through latent class analysis, and the impact of lifestyle on health status was examined using panel linear regression models. The research found that the lifestyle of the older adults can be segmented into three categories: relatively positive, mixed, and relatively negative. In 2018, the latent class probabilities of being relatively positive, mixed, and relatively negative were 45.40%, 2.69%, and 51.92%, respectively. It can be seen that the lifestyle structure of the older adults in China is complex and dominated by unhealthy lifestyle. Further, the lifestyle category has a significant positive effect on the health status of the older adults. For each improvement in lifestyle category, the chances of the older adults having better physical, mental, and self-assessed health increased by 11.2%, 3.6%, and 17.1%, respectively. This effect remained significant even after endogeneity analysis using the instrumental variable method, shedding light on the dynamic relationship between lifestyle and health among the older adults. Overall, a good lifestyle can improve the health status of the older adults. This indicates that the comprehensive effects of daily diet, personal hygiene, exercise, and leisure activities on the health of the older adults are significant, which is consistent with the existing research structure (35, 36). Unhealthy lifestyle has increased the mortality among older adults. Nearly 80% of deaths among older adults in China are caused by dietary risks (malnutrition or overnutrition), hypertension, smoking, elevated fasting blood sugar, air pollution (indoor and outdoor), and lack of exercise (1).

The rise in the aging population has emerged as a significant societal issue globally since the 20th century. As of 2019, individuals aged 65 and above constituted 9% of the world’s population (37), and nearly a hundred countries have entered an aging society. As projected by the United Nations, by 2050, the global population aged 65 and above will comprise 16% of the total population, surpassing 1.5 billion in total (38). Aging has established itself as an irreversible demographic trend in the near term. World report on aging and health emphasizes the need for health systems to gradually shift from disease based treatment models to comprehensive care models centered around the older adults. Guided by the above research findings, to foster healthy aging, efforts should aim to improve the health literacy of seniors, steer them away from unhealthy behaviors, and instill beneficial lifestyle habits.

Cockerham proposed the theory of healthy lifestyle, emphasizing that healthy lifestyle is influenced by both individual initiative and structural factors (9). The theory of healthy lifestyle points out that social structural factors such as class, gender, age, race or ethnicity, collectivity and living conditions can significantly affect an individual’s life opportunities. This dynamic interplay between life opportunities and life choices forges behavioral inclinations, evolving into habits—cognitive maps or perceptual schemas used to guide and evaluate choices, resulting in corresponding actions. When it comes to a healthy lifestyle, these behavioral patterns contribute to either positive or negative health routines; these health practices can, in turn, modify individuals’ behavioral tendencies. Consequently, nations should take action on health management (39), proactively devise relevant policies, furnish commendable health services and social environments for the older adults, and foster their physical and mental well-being along with their social adaptability. In particular, several key strategies should be implemented: Primarily, the role of grassroots health service organizations, such as community and township health centers, needs to be redefined. This involves shifting from the traditional “outpatient” approach to disease treatment toward a “health management” framework, concurrently authoring and disseminating guidelines for older adults’ health maintenance alongside regular diagnostic and treatment services. Additionally, health promotion and education for the older adults should be intensified through a blend of online and offline platforms. We should guide seniors to take proactive steps in enhancing their nutritional intake, ceasing smoking, moderating alcohol consumption, securing quality sleep, increasing physical activity, and participating actively in social endeavors. Next, enhancing public spaces, entertainment, and sports facilities is crucial to create an environment conducive to social interaction and recreational activities for the older adults. This involves actively organizing rich, diverse cultural and sports activities that benefit physical and mental health and are well-received by the older adults, thus promoting healthier leisure activities. Lastly, the health industry should be vigorously expanded. Health service-related businesses should be encouraged to align their services with the physical and mental characteristics of the older adults and their main health risk factors. A wide array of services such as health preservation, routine check-ups, consultation management, fitness programs, sports rehabilitation, health tourism, and more should be robustly promoted, facilitating a “comprehensive and full-cycle” management approach to the lifestyle of the older adults.

This study used data from a national survey, and the sample is representative. The results of this study provide evidence for understanding the older adults’ lifestyle and its impact on the health in Chinese. However, there are still some limitations of this study. Firstly, lifestyle is a broad and vaguely defined academic field, although this study has characterized the lifestyle of the older adults with 15 indicators from four aspects, the overall picture and historical changes of the older adults’ lifestyle are not adequately depicted, especially the lack of use of the internet and intelligent devices by the older adults. Secondly, due to data limitations, this study has limitations in measuring health, especially in using only one indicator to measure mental health. Therefore, further research can adopt more scientific measurement methods, such as Short Version of Center for Epidermal Studies Compression Scale (CESD-10), Self-reporting Inventory (SCL-90), Shortform UCLA Loneliness Scale (ULS-6), the geriatric compression scale (GDS), Self-Rating Anxiety Scale (SAS), Generalized Anxiety Disorder (GAD-7) and so on. Thirdly, lifestyles varies greatly among regions and are constantly changing. This study only used 4 years of data from China, and research on lifestyle and its impact on health needs to be continuously tracked.

## 5 Conclusion

This study indicated that the lifestyle structure of the older adults in China is complex and dominated by unhealthy lifestyle, and the lifestyle category has a significant positive effect on the health status of the older adults. In future, studies should focus on improving the health literacy of seniors, steering them away from unhealthy behaviors, and instilling beneficial lifestyle habits.

## Data availability statement

Publicly available datasets were analyzed in this study. This data can be found at: CLHLS (https://opendata.pku.edu.cn/file.xhtml?fileId=10357).

## Ethics statement

The studies involving humans were approved by the Institutional Review Board of the Peking University and Duke University. The studies were conducted in accordance with the local legislation and institutional requirements. The participants provided their written informed consent to participate in this study.

## Author contributions

XW: Conceptualization, Data curation, Writing – original draft, Writing – review & editing. HM: Data curation, Methodology, Software, Writing – review & editing. QJ: Data curation, Methodology, Visualization, Writing – review & editing.
